# Co-designing a Canadian adaptation of a lifestyle-oriented intervention aimed to improve daily functioning of individuals living with chronic pain: a multi-method study protocol of REVEAL(OT) Canada

**DOI:** 10.3389/fresc.2023.1281680

**Published:** 2023-11-24

**Authors:** J. Masse, S. S. Nielsen, J. R. Christensen, S. T. Skou, J. Côté, S. Saunders, É. Lagueux, A. Boulanger, J. Perez-Martinez, M. Lussier, M. G. Pagé

**Affiliations:** ^1^ Research Center of the Centre Hospitalier de l’Université de Montréal, Montreal, QC, Canada; ^2^ Department of Biomedical Sciences, Faculty of Medicine, Université de Montréal, Montreal, QC, Canada; ^3^ The Research and Implementation Unit PROgrez, Department of Physiotherapy and Occupational Therapy, Næstved-Slagelse-Ringsted Hospitals, Slagelse, Denmark; ^4^ Department of Regional Health Research, University of Southern Denmark, Odense, Denmark; ^5^ User Perspectives and Community-Based Interventions, Department of Public Health, University of Southern Denmark, Odense, Denmark; ^6^ Research Unit of General Practice, Department of Public Health, University of Southern Denmark, Odense, Denmark; ^7^ Research Unit of General Practice, Aarhus University, Aarhus, Denmark; ^8^ DRIVEN, Department of Sports Science and Clinical Biomechanics, University of Southern Denmark, Odense, Denmark; ^9^ Center for Muscle and Joint Health, Department of Sports Science and Clinical Biomechanics, Faculty of Health, University of Southern Denmark, Odense, Denmark; ^10^ Faculty of Nursing, Université de Montréal, Montreal, QC, Canada; ^11^ Faculty of Medicine and Health Sciences, School of Physical and Occupational Therapy, McGill University, Montreal, QC, Canada; ^12^ Faculty of Medicine and Health Sciences, School of Rehabilitation, Université de Sherbrooke, Sherbrooke, QC, Canada; ^13^ Research Center of the Centre Hospitalier Universitaire de Sherbrooke, Sherbrooke, QC, Canada; ^14^ Department of Anesthesiology and Pain Medicine, Faculty of Medicine, Université de Montréal, Montreal, QC, Canada; ^15^ Department of Anesthesia, McGill University Health Centre, Montreal, QC, Canada; ^16^ Alan Edwards Pain Management Unit, Montreal General Hospital, Montreal, QC, Canada; ^17^ Patient Partner, Centre Hospitalier de l'Université de Montréal, Montreal, QC, Canada; ^18^ Department of Psychology, Faculty of Arts and Sciences, Université de Montréal, Montreal, QC, Canada

**Keywords:** chronic pain, occupational therapy, REVEAL(OT), participatory research, implementation science

## Abstract

**Background:**

Living with chronic pain (CP) often implies major lifestyle changes, including modifications of daily routines and work. Surprisingly, few validated and effective interventions specifically target functional outcomes in this population. Redesign your Everyday Activities and Lifestyle with Occupational Therapy [REVEAL(OT)] is a lifestyle-oriented intervention led by occupational therapists that directly targets the daily functional challenges of living with CP. The intervention was initially developed and studied as an add-on to standard treatment delivered by Danish multidisciplinary specialized pain clinics. Adapting, implementing, and evaluating REVEAL(OT) within the Canadian healthcare system will contribute to broadening the scope of treatments offered in specialized pain clinics that do not yet include occupational therapy.

**Objective:**

The proposed study aims to define and refine REVEAL(OT)/CA with partners (authors of original intervention, people with lived experience, clinicians, managers).

**Methods:**

This participatory action research will use a multi-method design and follow the ORBIT model for developing behavioral treatments for chronic diseases. A process of co-construction with partners and an advisory committee will take place in two Montreal specialized pain clinics. It consists of two related work packages (WPs). In WP1, a first series of focus groups with partners (*n* = 86) and workshops with the advisory committee will be conducted to co-develop the hypothetical pathway describing intervention components and their potential mechanisms of action on targeted outcomes, as well as the first version of the adapted intervention manual. WP2 will co-refine REVEAL(OT)/CA by exploring its acceptability, feasibility and mechanisms of action through intervention deliveries (at least twice in each of two specialized pain clinics; *n* ≥ 60 patients) and focus groups and/or individual interviews with participating patients and partners. At the end of this study, the intervention manual will be generated both in French and English.

**Discussion:**

This study will set the stage for subsequent implementation and effectiveness assessment projects and be an important step towards the deployment of interventions aiming to improve engagement in meaningful daily activities among adults living with CP.

**Registration:**

OSF Registries, osf.io/8gksa. Registered 3 August 2023, https://osf.io/8gksa.

## Introduction

Chronic pain (CP), or pain that lasts more than 3 months (1), affects one in five Canadians (2), is associated with high disability (1, 3), and costs approximately CAN$40 billion each year when considering direct and indirect costs (4). CP frequently implies disruptions in their daily routines (5) and it is one of the chronic diseases associated with most years lived with disability (6) and occupational identity loss (7, 8). Despite the adverse human, societal, and economic consequences of CP (2, 9–13), it is often underdiagnosed and undertreated (14, 15). Biopsychosocial models (2, 4) are commonly endorsed theoretical understandings of the experience of pain, and multidisciplinary treatments are recognized as the gold standard for pain management (16, 17). Yet, current evidence does not portray a very optimistic picture, with most interventions only offering mild to moderate benefits (2, 4) and differential effects of treatment components being poorly documented (18). This can be illustrated by a recent study that showed that only 20% of individuals living with CP attending specialized pain clinics report clinically significant improvements in pain severity after 2 years (19).

In this context, recent reports from the Canadian Pain Task Force and the Action Plan for Pain in Canada highlight the need for innovative clinical models that can better treat or manage pain holistically (2, 4, 20). Individuals living with CP are concerned about the multiple occupational challenges they face on a day-to-day basis (8, 21, 22). Amongst 152 individuals living with musculoskeletal pain, 85% identified facing challenges associated with productivity (including paid/unpaid work, household management and play/school), 77% reported challenges associated with self-care (including all occupations related to personal care, functional mobility and community management) and 78% reported challenges associated with engaging in leisure activities (such as quiet recreation, active recreation and socialization) (23). These individuals believe that lifestyle is an important factor in health-related quality of life when living with CP and 92% of those entering a multidisciplinary pain treatment center report being motivated to change their lifestyle (24). Despite the high prevalence of occupational disturbances in this population and the need to address them, very few interventions within specialized pain clinics directly target occupational outcomes (9).

All these findings highlight the need for comprehensive programs promoting improved function and quality of life through occupational approaches (25, 26). According to the International Association for the Study of Pain [IASP] (27), occupational therapists are important members of specialized CP management clinics since they provide distinct value in the management of CP by directly targeting the person's engagement in significant occupations and by specifically using occupation itself as a medium for therapy (27–29). However, poor integration of occupational therapy within multidisciplinary care has been documented (30, 31) which might in part be due to a lack of training of occupational therapists in chronic pain management and poor visibility of the role occupational therapy can play in with this population (31). Lifestyle-oriented interventions, which put focus on facilitating client development and implementation of healthy routines and habits, have been studied recently (24, 32–34). It appears that, although pain intensity tends to remain statistically unchanged, engagement in meaningful activities was significantly improved, which is considered an important determinant of health, well-being and quality of life for individuals affected by CP (32, 34). Those interventions, however, were originally designed for chronic illnesses in general and were adapted to CP on a theoretical basis without involving collaborative process with partners. To our knowledge, very few experimental and quasi-experimental studies including occupational engagement in CP treatment for adult population are available.

Redesign your Everyday Activities and Lifestyle with Occupational Therapy [REVEAL(OT)], is an innovative intervention developed at the University of Southern Denmark and Region Zealand which has undergone three iteration phases to improve delivery, outcomes, and fit within specialized Danish pain clinics (24, 33, 35–37). Considering that several factors contributing to poorer mental and metabolic health and increased risks of chronicity (e.g., stress, physical activity, eating habits) are modifiable (38), addressing them could help break the vicious cycle of CP, poor functioning, and psychological distress. REVEAL(OT) proposes a combination of individual and group interventions over the course of 14 weeks. It is based on occupational therapy evidence about CP management through lifestyle changes, population-centered information on motivation for changing lifestyle, and contextual factors related to intervention delivery within a specialized tertiary care pain clinic. When added to usual care, REVEAL(OT) leads to satisfaction and improvement in a range of outcomes important to individuals living with CP, such as more effective activity pacing in real-life daily routines (33). A recent study has demonstrated REVEAL(OT) feasibility in a Danish pain clinic (35) by showing satisfactory program adherence and patients’ self-perceived relevance, timing and mode of delivery while retention and the fidelity of delivery needed improvement. This pre-post study detected a significant change in occupational performance and satisfaction (assessed by the Canadian Occupational Performance Measure, COPM). Change reached the minimal clinically important difference (MCID) for COPM performance in 14%, and for COPM satisfaction for 24% of the cases. Since it is specifically targeting occupational needs of individuals living with CP seen in specialized pain clinics (33), REVEAL(OT) was chosen for the current study over others with more effectiveness data but that were developed for other populations such as mental health (39, 40) or diabetes (41).

According to the Adapting Interventions to New Contexts (ADAPT) guidelines (42), it is often more efficient to adapt an existing intervention than to develop a new one. Identifying and exploring the impact of essential treatment components on targeted clinical outcomes is fundamental to the adaptation process (43). It is also crucial to involve partners since most health interventions are highly sensitive to the context in which they are delivered and it is important to ensure they meet the needs of the target population, they are deliverable by clinicians while considering organizational constraints (42). The proposed study aims to define and refine REVEAL(OT)/CA with partners (authors of original intervention, people with lived experience, clinicians, managers). This overall objective is operationalized in 4 specific objectives: (1) co-development of (a) the hypothetical pathway describing intervention components and their potential mechanisms of action on outcome and (b) the adapted intervention manual with partners (authors of original intervention, people with lived experience, clinicians, managers); (2) optimization of acceptability of intervention content and format, and feasibility of its delivery; (3) exploration of acceptability, feasibility and mechanisms of action of REVEAL(OT)/CA through initial delivery of the intervention; and (4) co-refinement of REVEAL(OT)/CA to generate a final manualized version prior to subsequent implementation and effectiveness assessment projects.

## Methods

### Overview

This participatory action research will use a multi-method study design to meet the stated objectives. Combining qualitative data about partners' appreciation of the intervention will drive outcomes used to explore its clinical effects, with an emphasis being placed on a democratic process in which all team members participate in research creation. An advisory committee will be formed of 2–3 patient-partners living with CP who have received care in specialized pain clinics, 2–3 clinicians from two pain clinics (coming from different professional backgrounds), 2 medical directors and 2 managers (at least 1 from each clinic), 3 international researchers which are also authors of REVEAL(OT) and 3 local researchers (with complementary research expertises). The composition of the committee may change over time depending on the expertise required, the availability of each member and the evolution of the project. The ORBIT model for developing behavioral treatments to prevent and/or manage chronic disease (43–46) will guide the methodology. This model, which is informed by the meticulous process of drug development, is based on a large consensus between renowed scientific committees including the Obesity-Related Behavioral Intervention Trials (ORBIT) consortium. It proposes a flexible and iterative progressive process, in which prespecified clinically significant milestones will guide forward or backward movements between each phase in order to develop an intervention and utimatly determine its effectiveness. The present study will carry out Phase Ia (*Define the intervention*) and Ib (*Refine the intervention*) of the ORBIT model.

### Study design

**Figure 1:**
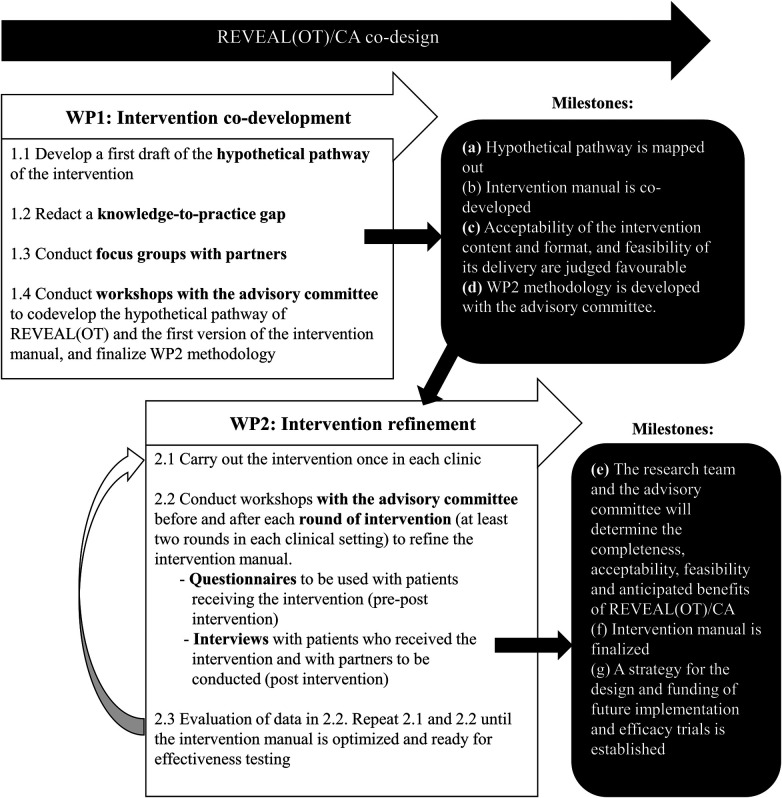
A visual model of the study design and work packages that will be executed.

The current project consists of two related work packages (WPs) that aim to codesign a Canadian adaptation of REVEAL(OT) in two Montreal specialized pain clinics. WP1 will provide the scientific basis to define key features of the intervention, co-develop the adaptation of REVEAL(OT)'s manual, and theoritically assess acceptability and feasibility of its content and format in collaboration with partners and the advisory committee (Obj. 1 and 2). This will be achieved by conducting intervention development focus groups (*n* = 86 partners) and workshops. Partners recruited for WP1 (intervention development) will include patients, clinicians and managers from both pain clinics. In order to refine the intervention, WP2 will consist in delivery of the intervention (at least twice in each of two specialized pain clinics; *n* ≥ 60 patients) followed by interviews with patients who received the intervention and WP1 partners (or new ones if necessary). This step will thus explore its acceptability, feasibility and mechanisms of action both quantitatively and qualitatively (Obj. 3) in order to achieve an improved version of REVEAL(OT)/CA which will fit the two targeted clinical settings and plan future studies (Obj. 4). A visual model of the two WPs is presented in Figure 1 and the protocol for each WP is described in detail below.

#### Work package 1: definition of REVEAL(OT)/CA

To begin, we will co-define the key features of the intervention based on the following steps (43, 44, 47–50):

(1.1) develop a first draft of the hypothetical pathway of the intervention using the English translation of the original intervention and reviewing scientific knowledge and clinical guidelines about lifestyle-oriented interventions in CP management,
(1.2) identify knowledge-to-practice gaps taking into consideration organizational practices, policies and procedures of the two targeted clinical settings,
(1.3) conduct intervention development focus groups with partners using the hypothetical pathway and the synthesis of the knowledge-to-practice gap as the focus of discussion, to inform the adaptation of the intervention and to assess the theoretical acceptability of the intervention content and format, and feasibility of its delivery, and
(1.4) conduct workshops with the advisory committee to co-develop the hypothetical pathway of REVEAL(OT), first version of REVEAL(OT)/CA manual (in French and in English), and WP2 methodology.

##### Participants and recruitment for the intervention development focus groups

Eligible participants will be (a) those living with pain for more than 3 months, speaking French and/or English, and who have received treatment at one of the two participating pain clinics within the past 12 months; and (b) health care providers working with individuals with CP at the participating pain clinics for more than 12 months, and managers officially involved in administrative and/or managerial tasks involving the pain clinics. A purposive maximum variation sampling will ensure that we include the perspectives related to sex and gender (patients, clinicians, and managers), socio-economic level (patients), urban/rural living (patients), care paths (patients) and healthcare disciplines (clinicians). Patients will be recruited via posters at each clinic site and from invitations sent electronically or in person by the health care team. Clinicians and managers will be invited electronically or in person via emails and posters. The institution's social medias will also be used to disseminate the recruitment posters as needed. A member of the research team will contact interested individuals to assess eligibility and obtain written consent.

##### Data collection

A brief sociodemographic questionnaire will be completed by all participants using Research Electronic Data Capture (REDCap). Patients, clinicians, and managers will be invited to discuss service gaps in CP interventions regarding occupational needs, and comment on the proposed intervention content and format. To do so, in-person and/or online semi-structured focus groups lasting approximately 90 min will be conducted separately for patients (2–3 groups of 6–8 patients/clinic; *n *≈ 24 at each site) and clinicians/managers (1–2 groups of 6–8 clinicians from various backgrounds and 2 managers/clinic; *n *≈ 18 at each site) to facilitate sharing (51). The interview guide will be informed by Proctor et al.'s conceptual framework and taxonomy of implementation outcomes (52, 53) and will structure questions around the implementation outcomes identified in this framework including exploration of acceptability, adoption, appropriateness, feasibility, fidelity, implementation cost and penetration. These focus groups will be audio-recorded and transcribed verbatim. One of the core advisory committee members will be assigned for taking minutes and synthesize overall group discussions. These documents will also serve as data to design the adapted version of the intervention.

##### Sample size and feasibility of recruitment

Approximately 20 patients, ten clinicians and two managers will be recruited in each clinic. Final sample size will be guided by the concept of information power (51, 54, 55), meaning that it will depend on the amount of relevant information obtained as we go on with the interviews.

##### Data analysis

A two-step framework-guided *rapid analysis* will first be conducted in order to produce rigorous and rapid results to inform ongoing co-development process of the hypothetical pathway and the intervention manual (56–58) followed by a reflexive thematic analysis in order to deepen our understanding of participants point of view about implementation outcomes. *Step 1.* Audio recordings and/or verbatim transcripts will first be summarized using a structured template based on Proctor et al.'s taxonomy (52, 53). The template summary table will be developed by one researcher tested by a subgroup of researchers with a single transcript, and then, reviewed and modified. A review of the summaries will be conducted by the lead researcher to ensure consistency in the data being recorded and modifications will be done if necessary (56). *Step 2*. Summaries will subsequently be consolidated into a matrix (MS Excel document) by participant type (patients vs. clinicians and managers) to identify frequently occurring themes about participants' point of view including illustrative quotes. Within each theme of the structure template (lines) sub-themes (columns) will summarize satisfactory aspects of the hypothetical pathway and intervention manual and areas for improvements while allowing comparison across groups (one tab per group) (56, 59–64). *Reflexive thematic analysis.* After the hypothetical pathway is mapped out and the first draft of the intervention manual is co-developed with the advisory committee, a six-phase approach to reflexive thematic analysis (59, 65) will be performed in order to provide a richer and more detailed account of data obtained from the focus groups (56, 59, 60, 65, 66). These phases will include familiarization with the data by research team members to gain initial insight of the data through sharing perspectives, generating codes using primarily a deductive approach based on Proctor et al.'s taxonomy (52, 53) while allowing for inductive analysis also to make sense of data, searching, reviewing and defining main themes, and producing the final report. To do so, researchers will revisit the research question, notes from the familiarization phase, lists of codes and theme definitions, while making connections with existing research and literature.

Data validation strategies will include participants' input (three participants from different backgrounds will be re-contacted) and deviant cases (cases not fitting conclusions and account for these differences) (67, 68). Divergent perspectives will be considered in order to broaden our results and discuss the strengths and limits of our findings (69).

##### Milestones

At the end of WP1, (a) the hypothetical pathway describing the components of the intervention and their potential mechanisms of action on the targeted outcomes, as well as the (b) first version of REVEAL(OT)/CA intervention manual will have been co-developed with the advisory committee; (c) acceptability of the intervention content and format, and feasibility of its delivery will be judged favourable by partners and the advisory committee, and (d) WP2 methodology will have been developed with the advisory committee.

#### Work package 2: refinement of REVEAL(OT)/CA

Based on WP1 results, we will:

(2.1) run the adapted intervention at least twice in each clinical setting to explore its acceptability, tolerability, acceptability, feasibility and effects (quantitatively and qualitatively), and ultimately achieve an improved version of REVEAL(OT)/CA which will fit the two targeted clinical settings and plan future studies,
(2.2) workshops with the advisory committee before and after each round of intervention will be conducted and
(2.3) analyzed to co-develop and improve the intervention manual. This sequence will be repeated until the intervention manual is optimized and ready for effectiveness testing.

This second phase of the study refers to phase Ib of the ORBIT model and will use a mixed methods approach including a fractional factorial design (70–72). By manipulating specific components of an intervention as independent variables (or factor), fractional factorial designs are efficient ways to reach excellent statistical power even with relatively few participants which are used to estimate the effects of each component of the intervention and to assess their interactions (70, 71, 73, 74). The most important components, identified in WP1, will be examined using this fractional factorial design. An iterative process between intervention delivery with data collection, and refinement of the manual will take place until WP2 milestones are achieved.

##### Participants and recruitment

###### Patients

Eligible patients will be adults diagnosed with a CP condition who understand spoken and written French and/or English, have access to the Internet and a virtual platform. Individuals with cancer, active suicidal thoughts, substance misuse, severe psychiatric diagnoses such as psychoses, or those currently completing other intensive group or individual interventions (e.g., group psychotherapy) will be excluded. Patients will be referred for the intervention by one of their clinicians at the pain clinic based on their functional deficit in at least one occupational domain (self-care, leisure, productivity; regardless of severity), and this referral could be done at any time point in their treatment path. If interested, they will meet with a research assistant to confirm eligibility through in-person meeting or phone call, and written consent will be obtained.

###### Clinicians and managers

Clinicians of the participating pain clinics who have had at least one clinical encounter with a patient participating in the program and managers consulted in WP1 will also be eligible. They will be invited electronically or in person by a member of the research team and written consent will be obtained.

##### Interventional methods

REVEAL(OT)/CA will be run at least twice at each site. The intervention will be co-led at both sites by the lead occupational therapist and one trained occupational therapist that will be recruited from within the hospital setting (or from the community of practice if not available internally) to start building capacity. The latest version of the intervention manual and script protocols or guidelines to support the conduct of each treatment session will be used to enable intervention delivery as planned (75).

##### Data collection

Once determined eligible, recruited patients will provide written consent and be asked to complete a sociodemographic questionnaire followed by a battery of self-report questionnaires within 2 weeks prior to the start of the intervention using the online data capture software (REDCap). These preliminary questionnaires will measure functioning, psychological state, and pain (T0; see Table 1). Final choice of questionnaires will be done in close collaboration with the advisory committee during WP1 workshops. Fidelity to the intervention delivery will be monitored at each session using a pre-established content and behavioral checklist based on the National Institutes of Health Behavior Change Consortium treatment fidelity recommendations and REVEAL(OT)/CA theory and structure (75, 88, 89). This checklist will be completed by clinicians who provide the intervention and research team members who will observe approximately 25% of intervention sessions (or review audio-taped intervention sessions) followed by discussions to correct any deviations in intervention delivery (75, 90–92). Within 2 weeks after the final session, participants (patients) will be invited to complete end-of-treatment measures (T1). Adverse effects (such as unpleasant experiences such as discomfort and cognitive load) (93, 94), as well as process evaluation outcomes (such as information and treatment load) which have been documented in a previous REVEAL(OT) feasibility study (33) will also be collected through a brief survey at the end of each session.

**Table 1:** Description of self-report measures and time of administration.

Measures	T0	T1
Sociodemographic questionnaire will include information about age, gender identity, ethnicity, education level, marital status, work status, source of income and disability benefits, first 3 digits of postal code	X	
Occupational engagement: the engagement in meaningful activities survey (76, 77) is a 12-item self-report measure assessing an individual's perceived engagement in meaningful activities	X	X
Quality of life: the short-form health survey V2 (SF-12v2) (78) is a 12-item questionnaire aimed at measuring quality of life, through two norm-based summary domains: physical and health-related quality of life	X	X
Pain characteristics: body map, circumstances surrounding pain onset and pain duration (79)	X	
Pain intensity: the numeric rating scale (80) is a single 0–10 measure that is used to measure different aspects of their pain experience (current, average, worst pain intensity over the past 7 days; average pain unpleasantness over the past 7 days)	X	X
Pain interference: the brief pain inventory (81, 82) is a 7-item scale that measures the extent to which pain interferes with daily activities. Each item is ranked on a scale from 0 (no interference) to 10 (completely interferes)	X	X
Psychological distress: the patient health questionnaire-4 (PHQ-4) (83) is 2 4-item scale briefly screening for anxiety and depressive symptoms	X	X
Self-efficacy: the pain self-efficacy questionnaire (84) is a 10-item measure of how confident one is at coping with pain	X	X
Catastrophizing: the six-item short-form pain catastrophizing scale (85, 86) is a 13-item scale assessing levels of pain rumination, amplification and feelings of helplessness in the presence of pain	X	X
Satisfaction: the pain program satisfaction questionnaire (87), is an 11-item scale measuring the extent to which individuals are satisfied with the program, using ranked items (1 = not at all, 4 = definitely/extremely satisfied)		X

All patients who will have received the intervention, their clinicians, and managers will be invited to participate in semi-structured interviews to document their point of view regarding acceptability of format, content and mode of delivery of the intervention, as well as perceived barriers and benefits, areas for improvement and factors that would influence future implementation of the intervention. These interviews will be conducted online (via Zoom) or in-person and will be audio-recorded and transcribed verbatim. Interview guide will be co-constructed in WP1.

##### Sample size and feasibility of recruitment

###### Intervention delivery

Considering that up to 15 patients will be enrolled per group, and considering a 25% attrition rate, it is anticipated that approximately 11 patients would complete the intervention and outcome measures per group for each round of intervention. A sample size of 40 patients receiving the intervention would be sufficient to conduct feasibility assessment in health service intervention (95). There will be a pool of more than 100 patients per clinic per month. Considering that the groups will be run consecutively and not in parallel, we anticipate that we will be able to recruit the 15 patients for each group within a 1-month period prior to each group starting.

###### Post-intervention interviews

At least four patients, two clinicians and two managers (one form each clinic for each category of participants) will be interviewed. As in WP1, final sample size will be guided by the concept of information power (54, 55).

##### Quantitative data analyses

Using descriptive statistics (mean, standard deviation, median, minimum and maximum), we will compare sociodemographic data of participants to those of the clinics to examine representativeness of patients exposed to the intervention. Exploratory data analysis will use paired t-tests using pre/post-tests results as all variables are continuous. All results—positive, negative, and inconclusive—will be reported. Analyses will be conducted in R version 4.1.2 or higher.

##### Qualitative data analysis

Following the same methodology described in WP1, a *rapid analysis* will again be conducted (51) in order to capture the main preoccupations of our partners (patients who will have received the intervention, their clinicians, and managers consulted in WP1) about what worked well and opportunities for improvement (56, 63). A *reflexive thematic analysis* will follow in order to deepen our understanding of participants' experience (59, 65). Rapid analysis will now focus on both planned (i.e., changes to be carried out before introducing the intervention) and responsive (i.e., intentional changes resulting from emerging contextual issues during delivery) adaptations of the intervention content and format (42) to achieve an optimal fit between REVEAL(OT) and its new context. Using a similar approach as in WP1, we will use iterative cycles in which workshops with the advisory committee will be conducted to present results after each delivery of REVEL(OT)/CA and pursue its refinement considering quantitative and qualitative findings. In order to capture all aspects of participants experiences with a more nuanced and detailed level, a reflexive thematic analysis will subsequently be performed (59, 60, 65) as explained in WP1. Again, data validation strategies will be used (67, 68) and divergent perspectives will be considered (69). General principles of analysis will include reading through data, memo writing, coding, identifying themes, and interrelating themes. Two cycles of coding will be used (open coding followed by axial coding to examine relationships between codes) (59, 60). WP2 data analysis will lead to a revised version of REVEAL(OT)/CA and an overall strategy for future studies.

##### Milestones

At the end of this second phase of the study, the research team and the advisory committee will have determined the completeness, acceptability, feasibility and anticipated benefits of REVEAL(OT)/CA, finalized the intervention manual, and established a strategy for the design and funding of a future implementation and efficacy trials.

## Discussion

Given the heavy individual, societal and economic impact of CP, and the importance of developing integrative approaches to better manage and treat CP (96), this research project will contribute to fulfilling several gaps identified in the literature by meeting the occupational needs of patients within the Canadian healthcare system. By doing so, it will support rehabilitation as one of the key components of CP management while directly building capacity among occupational therapists through the development of empirically-based effective lifestyle-oriented interventions in chronic pain management. The study methodology uses rigorous intervention development and conceptual models which put an emphasis on partner involvement at all phases of the study to reach an optimal fit between an intervention and its new context (42, 46, 47). Following this iterative process, REVEAL(OT)/CA will be designed to fit two Montreal specialized pain clinics that do not yet include occupational approaches. Not only will this study set the stage for a subsequent randomized controlled trial to test REVEAL(OT)/CA's effectiveness and later implementation, it will also increase visibility for the role and unique contribution of occupational therapy in complementarity with CP management treatments already offered.
